# Structures to complement the archaeo-eukaryotic primases catalytic cycle description: What's next?

**DOI:** 10.1016/j.csbj.2015.04.006

**Published:** 2015-05-02

**Authors:** Julien Boudet, Jean-Christophe Devillier, Frédéric H.-T. Allain, Georg Lipps

**Affiliations:** a Department of Biology, Institute of Molecular Biology and Biophysics, ETH Zürich, 8093 Zürich, Switzerland; b University of Applied Sciences and Arts Northwestern Switzerland, Gründenstrasse 40, 4132 Muttenz, Switzerland

**Keywords:** Primases, High-resolution structures, Catalysis, Dinucleotide formation, DNA template, Primer synthesis

## Abstract

DNA replication is a crucial stage in the transfer of genetic information from parent to daughter cells. This mechanism involves multiple proteins with one key player being the primase. Primases are single-stranded DNA dependent RNA polymerases. On the leading strand, they synthesize the primer once allowing DNA elongation while on the lagging strand primers are generated repeatedly (Okazaki fragments). Primases have the unique ability to create the first phosphodiester bond yielding a dinucleotide which is initially elongated by primases and then by DNA polymerases.

Primase activity has been studied in the last decades but the detailed molecular steps explaining some unique features remain unclear. High-resolution structures of free and bound primases domains have brought significant insights in the understanding of the primase reaction cycle. Here, we give a short review of the structural work conducted in the field of archaeo-eukaryotic primases and we underline the missing “pictures” of the active forms of the enzyme which are of major interest. We organized our analysis with respect to the progression through the catalytic pathway.

## 1 Primases are required for replication

In all living organisms, the semi-conservative duplication of DNA is a crucial step ensuring the transmission of genetic information through the generations. DNA replication is a tightly regulated process involving many molecular partners and discrete interactions contribute to coordination and replication efficiency [1]. Among the components required for DNA duplication, helicases unwind the anti-parallel strands, primases synthesize a short RNA primer complementary to the continuous single-stranded DNA strands and finally DNA polymerases carry out the bulk of replication by elongating the primers. Since DNA polymerases exclusively polymerize in the 5′ to 3′ direction, the leading strand is continuously duplicated while replication of the lagging strand necessitates multiple DNA-producing subcomplexes [2]. Only primases can synthesize template-annealed oligoribonucleotide primers allowing for DNA polymerase-mediated elongation. The newly synthesized primers are later removed by an RNase, substituted by oligodeoxyribonucleotides and ligated to preserve chain continuity. One should mention that some archaeal primases can incorporate deoxyribonucleotides in the primer [3].

### 1.1 Primases

Primases are grouped into two major classes: the bacterial/bacteriophages and the eukaryotic/archaea enzymes [4,5]. Viral primases potentially constitute an additional group [6]. The bacterial and archaeo-eukaryotic primase groups differ in both their functional assembly and structural organization. The bacterial and bacteriophage primase form a protein complex with the replicative helicase of superfamily 4. These helicases encircle the single-stranded DNA of the lagging strand of the replication fork and consequently move in the 5′ to 3′ direction. In some bacteriophages' primase and helicase are even encoded in a single protein, *e.g.* gp4 of the T7 phage, underscoring the functional interplay of these two proteins at the lagging strand. In contrast the replicative helicases of eukaryotes and archaea, the MCM proteins, belong to helicase superfamily 3 and appear to encircle double-stranded DNA in its unwinding process. The MCM proteins are not associated with the primases. Rather the archaeo-eukaryotic primases associate the DNA polymerase alpha. Here, we review only archaeo-eukaryotic enzymes; investigations on prokaryotic/phages primases can be found elsewhere [2].

### 1.2 Features of archaeo-eukaryotic primases

**Fig. 1:**
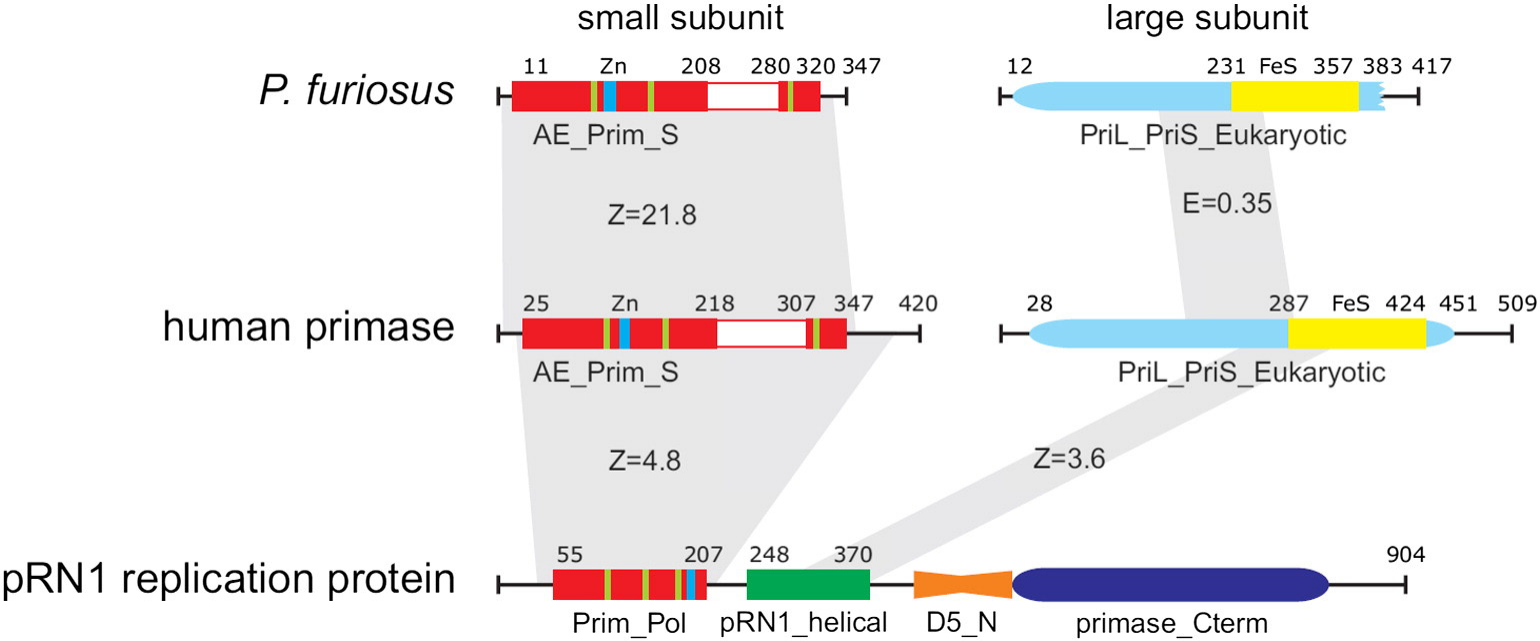
Domain organization and structural comparison of primases. We compared the domain organization from *Pyrococcus furiosus* primase (small subunit: 1G71, large subunit: no structure available), human primase (4RR2) and the pRN1 multifunctional replication protein ORF904 (partial structure from amino acids 40–370, 3MIM). The domains of the respective proteins were determined with RPS-Blast and HHpred against the conserved domain database. Yellow rectangles indicate the position of the Fe–S cluster and cyan rectangles show the position of the zinc binding region. The white rectangles within the small primase subunits correspond to the unrelated helical domains which interrupt the prim fold. Green lines define the positions of three structurally highly conserved β-strands of the prim fold. The first β-strand harbors two conserved acidic residues, the second contains a highly conserved histidine and the last one is the flange running perpendicular to the other strands. Gray trapezoids highlight structural similarity as detected by DALI or BLAST if structural information is missing. The quality of the alignment is given with the Z-score or the E-value respectively. Numbers above each picture designate the limits of the domain borders and the length of the respective proteins. Proteins are drawn to scale.

Archaeal and eukaryotic primases share important structural similarities and the existence of a common ancestor has been repeatedly suggested (Fig. 1) [7]. Several studies [2,8] on eukaryotic primases distinguished two independently-folded parts required for primer-mediated DNA replication initiation and elongation. The small catalytic (p49 or PriS) and the large regulatory subunits (p58 or PriL) of eukaryotic primases interact tightly (Kd of 0.46 nM between PriS and PriL-NTD [9]) to form a protein complex with the 180-kDa DNA polymerase α by interacting with the p70 polymerase B subunit. In archaea the primases are also heterodimeric but do not appear to be associated with a DNA polymerase as in eukaryotes. So far the primases of only a few model organisms have been studied and it is clear that these enzymes have the same core function of synthesizing primers. Nevertheless differences in quaternary structure (*e.g.* association with other proteins of the replication fork), nucleotide preference (*e.g.* some archaeal primases may also use deoxynucleotides), primer length and template specificity are key parameters of catalysis. In addition, various local and global structural rearrangements may stabilize transition states.

**Fig. 2:**
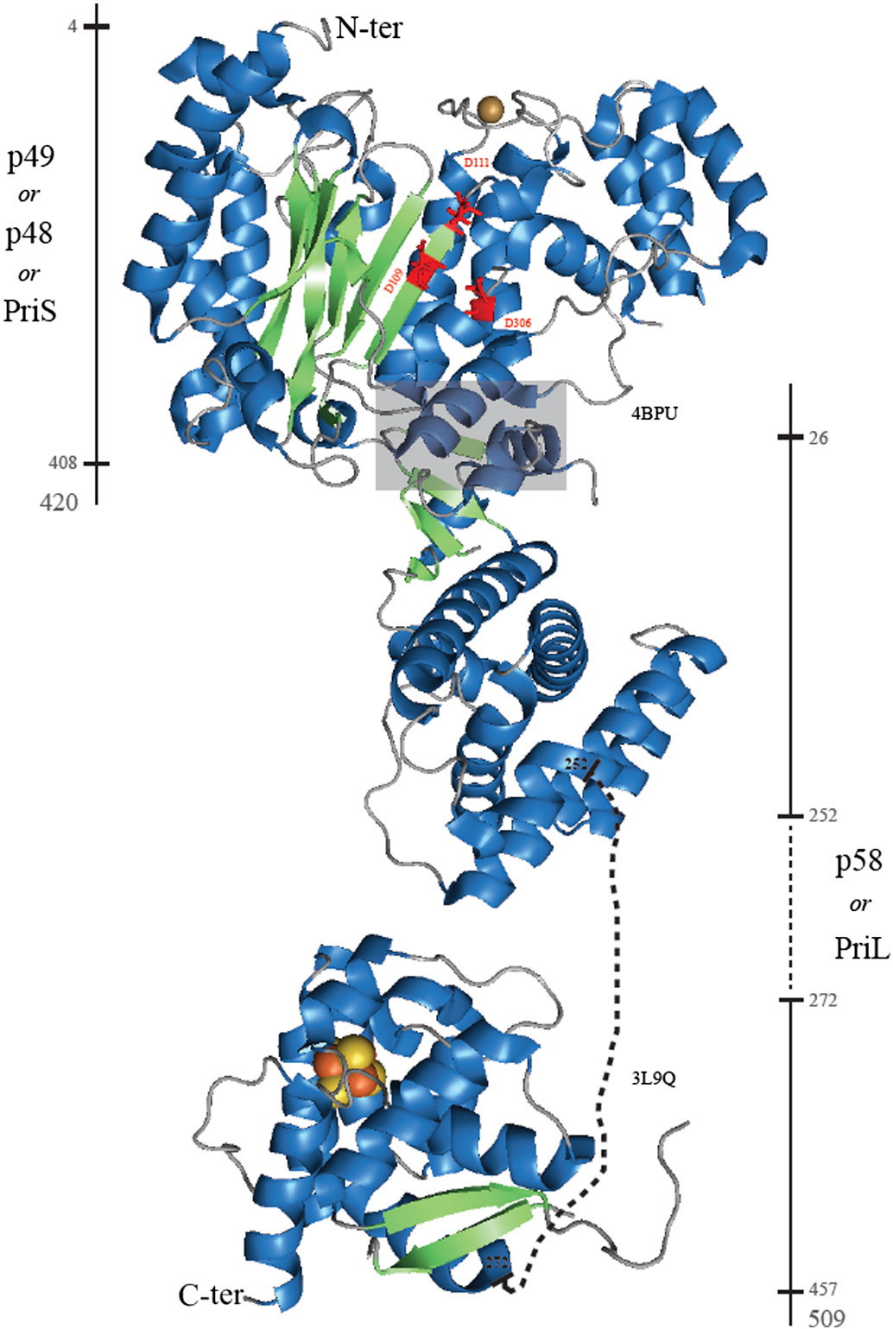
Structural organization of the human primase reconstituted from two independently solved structures. We used the structure of the unliganded small subunit, PriS, complexed with the N-terminal half of the large subunit, PriL, (4BPU) in combination with the structure of the iron–sulfur bearing C-terminal half which was solved independently (3L9Q). One should note that a complete structure of the heterodimeric human primase is now available (4RR2). Orange and yellow spheres of the metal cluster correspond to iron and sulfur atoms respectively. The small subunit harbors the active site of primer synthesis with the triad of aspartate residues (D109, [D111 and D306] D111, D306) shown with red sticks. This β-sheet constitutes the RRM (RNA recognition motif). The brown sphere is a zinc ion. A dashed line represents the twenty missing residues connecting the independently solved structures of the N- and the C-ter part of the PriL domain. Solid lines and numbers surrounding the human primase define the boundaries of the structural elements. Residues 26 to 252 refer to the α-helical subdomain. The gray-shaded box highlights the interaction interface between PriS and PriL. Two structural parts cannot be detected in the electron density of the PriS domain (residues 280 to 289 and residues 358 to 385) concomitantly to four elements in the PriL domain (residues 85 to 93, residues 174 to 182, residues 332 to 345 and residues 354 to 358). α-helices, β-strands and loops are depicted in blue, green and gray respectively.

For archaeo-eukaryotic primases, the catalytic core classically adopts a fold related to the RNA recognition motif (RRM) [10], see Figs. 2 and 8 and [11]. This part contains a highly conserved triad of acidic residues and a histidine residue. These residues have been reported to be critical for nucleotide polymerization and may be involved in the two-metal ion catalytic mechanism [12] involving either magnesium or manganese ions [3,13].

### 1.3 Priming steps

**Fig. 3:**
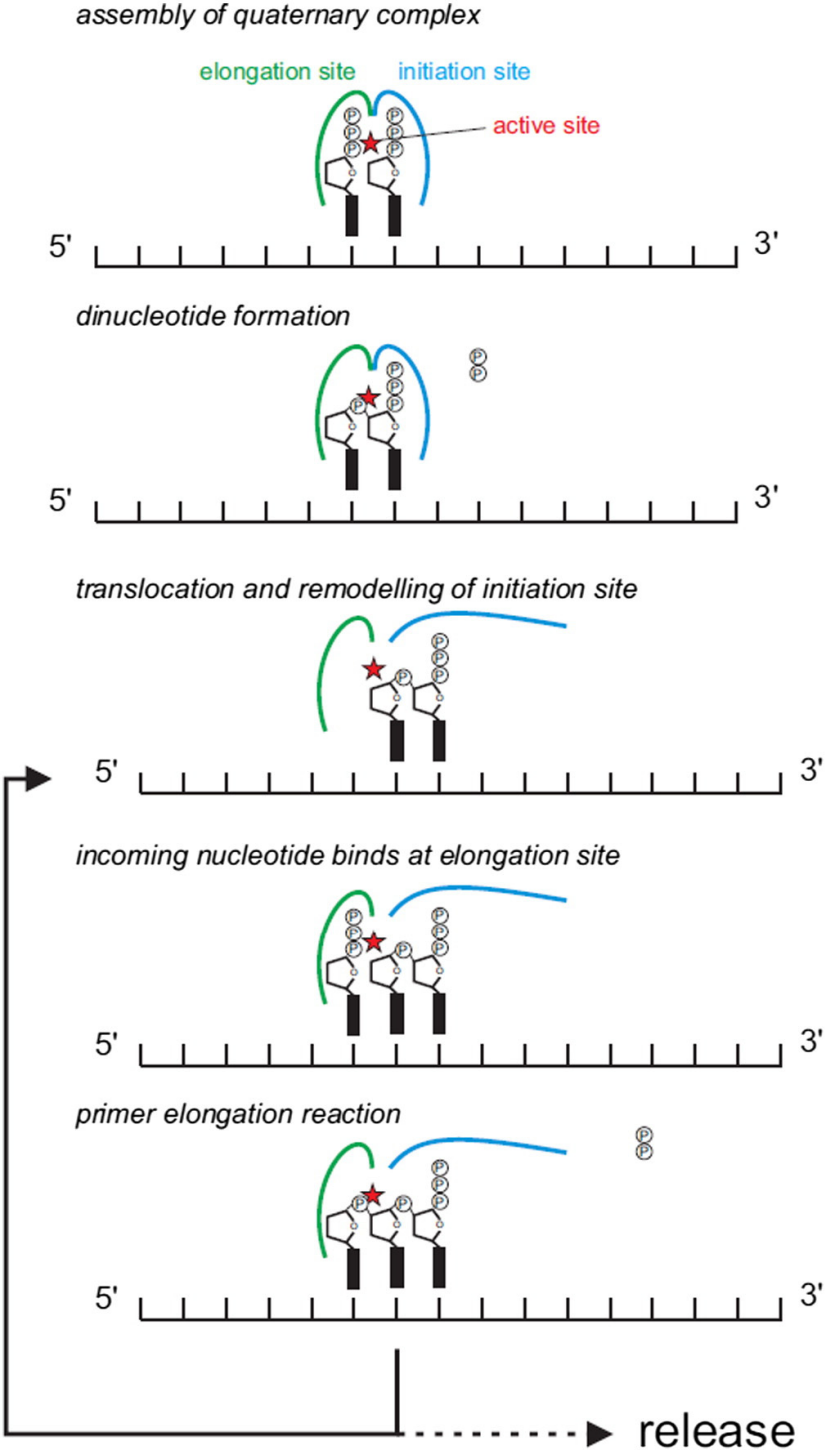
Simplified view of the dinucleotide formation step and its repositioning at the active site after the quaternary complex assembly. The thick black rectangles correspond to the base rings of the initiating and the incoming nucleotides. The “P” letter refers to phosphate groups. The horizontal thin line (5′ to 3′) with its small vertical bars represents the template DNA sugar-phosphate backbone and its bases respectively.

Extensive biochemical analyses combined with recent structural results have significantly improved our knowledge of molecular events characterizing the catalytic cycle. Therefore, one can differentiate four basic steps: NTP binding, template binding, di-nucleotide formation followed by repositioning at the active site to allow for subsequent primer extension (Fig. 3).

Primer synthesis occurs in preferred sites on the DNA template. *In vitro*, eukaryotic primases have only minimal specificity requirements and studies demonstrated preferences for pyrimidine-rich templates [14]. Furthermore, the 5′ nucleotide of the eukaryotic primers is often a purine. However, *in vivo*, DNA sequences appear to be less important for primase site selection as this process is guided by protein assemblies which control primase anchoring [15]. In contrast, some archaeal primases initiate synthesis at specific tri-nucleotide motifs within the template [16] while some others are less sequence-dependent [17]. The type and the concentration of NTPs may also influence primase site selection *in vitro* [18,19].

After template and NTP binding, primer synthesis comprises two kinetically distinct stages: the formation of the first phosphodiester bond and the elongation of the primer. For dinucleotide formation, two distinct nucleotide binding sites are required (Fig. 3): a site for the initiating nucleotide which will become the phosphorylated 5′ end of the primer, and the elongation site which will harbor the nucleotide which is hydrolyzed and linked to the 3′ end of the initiating nucleotide. After dinucleotide formation, the dinucleotide has to reposition within the active site in order to allow the new incoming nucleotide to occupy the elongation site. It is currently unclear whether this translocated dinucleotide is partly accommodated in the initiation site or whether it is stabilized by other parts of the holoenzyme.

Remarkably, eukaryotic primases generate primers of constant length (~ 10 nucleotides) and multiples thereof, but this ability to ‘count’ might not be shared with archaea [7]. Lastly, in eukaryotes, the newly synthesized primer is transferred to the DNA polymerase α for further extension. However the molecular details of this hand-over event remain elusive.

### 1.4 A valuable structural input

**Fig. 4:**
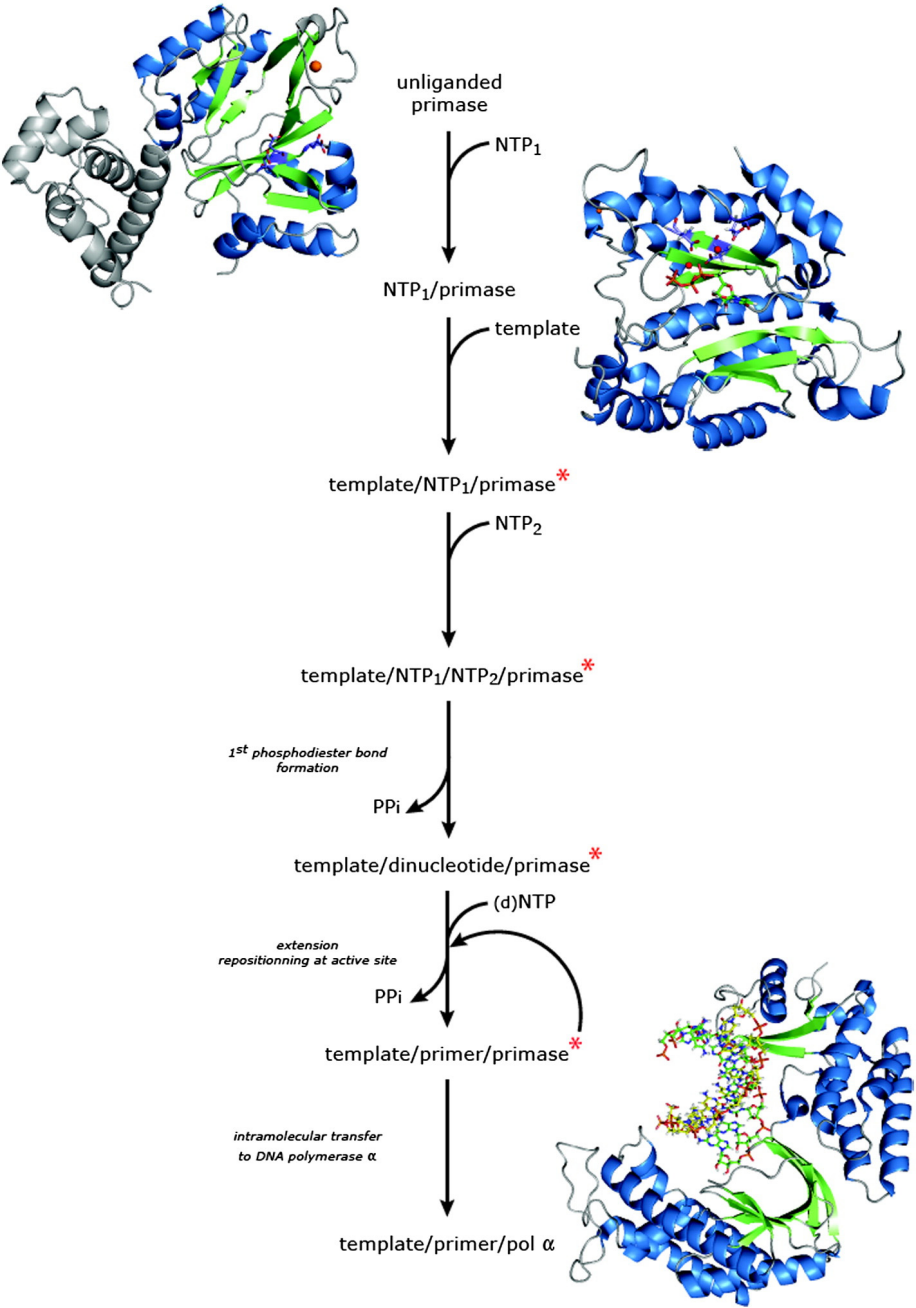
Available and expected relevant structural snapshots of the primase action. We divided the catalytic process into 6 discrete steps and indicated the respective partners (substrates and products) involved in the different complexes. Note that the order of substrate binding is not known. The structure of the primase in its free state (*e.g.* pRN1 replication enzyme, PDB ID 3M1M) and the structure of the nucleotide bound primase (*e.g.* UTP-bound catalytic core of the human p49 subunit, PDB ID 4BPW) illustrate the early stages of the reaction. NTP_1_ corresponds to the incoming nucleotide while NTP_2_ is used to describe the nucleotide inserted in the hypothesized initiation site. The highly conserved acidic residues are depicted in purple, red, blue and white sticks for C, O, N and H atoms respectively. The UTP molecule is shown with green, red, blue, orange and white sticks for C, O, N, P and H atoms respectively. Zinc and magnesium ions are displayed with orange and red spheres respectively. Red stars indicate the missing three-dimensional structures considered for their high value in the understanding of the whole enzymatic reaction (see also Concluding remarks: What is missing?). For clarity, only residues 848 to 1242 of the yeast polymerase α structure (PDB ID 4FXD) are shown. In the template/primer/polymerase α ternary complex the DNA and the RNA primers are in stick representation with C atoms in yellow and green respectively (the same color code as UTP was used for O, N, P and H atoms). In some archaeal organisms [16] and very inefficiently in the yeast primase [59] dNTPs can be integrated in the primer (small d in brackets).

Structural studies focusing on primases are not trivial. The number and heterogeneity of the replication machinery components has hampered standard solution-state NMR and crystallogenesis-based advances. In addition, the transient nature of interactions between substrates, protein effectors and products makes the isolation of discrete complexes challenging. Despite these limitations, structural findings in the last decade have substantially refined priming pictures at the atomic scale (for an overview see Table 1, Fig. 4).

First, the unliganded primase is examined. Then, we present a structure-based analysis of four major events: template binding, NTP binding, dinucleotide formation and primer extension. Furthermore, we relate our findings to complementary structures in order to provide a comprehensive understanding of the enzymatic process. Finally, we discuss some primase inhibitors and interests for medical applications.

## 2 Structural organization of the unliganded enzyme

We strategically focus this analysis on four structural features of the archaeo-eukaryotic primases. Beyond the oligonucleotide synthesis unit (catalytic site), the structural zinc motif, the iron–sulfur cluster and α-rich domains (Fig. 1) are essential to allow primer synthesis. Successful structural studies in the last decade often required truncated version of the protein for crystal formation [20–23] attesting to the heterogeneity and intrinsic dynamic properties of the enzyme.

### 2.1 The catalytic site

The catalytic site of archaea/eukaryotic primases adopts a mixed α/β architecture commonly called the ‘prim’ fold. Typically, external α-helices flanking two β-sheets delimit the central catalytic cleft (Fig. 2) lined by conserved residues. The number and the length of these secondary structures change between primases [21,23–25]. In average, six helices are packed against the β-sheets. To date, all the catalytic domain structures of archaea/eukaryotic primases showed an invariant cluster of acidic residues. A triad of aspartates is reported in six published structures [12,20–22,25,26] whereas one glutamate and two aspartates are present in the *Sulfolobus islandicus* pRN1 prim/pol domain. In nucleic acid polymerases these types of residues are also required to chelate divalent metal ions [27] which suggests that primases could use a similar catalytic process. Recent results confirm the importance of several basic amino acids located in the active site [25]. The eukaryotic SGXRG motif and conserved histidines are involved in direct, metal ion-mediated and stabilizing interactions with the nucleotide triphosphate (details in the ‘Nucleotide binding’ section). Studies combining alanine substitution, affinity measurements and primase activity assays have revealed an intricate network of conserved residues necessary for nucleotide [25] and DNA binding [23]. Modifications of the amino acids coordinating the zinc ion have produced intriguing results [22] that current structures of archaeo-eukaryotic primases cannot fully explain since no direct interactions with substrates and/or products have been reported yet. We believe it is relevant to mention all the available information about this structural motif.

### 2.2 The structural zinc motif

Except for the RepB′ enzyme of the RSF1010 plasmid [26], a zinc ion is present in all the reported archaeo-eukaryotic primases and stabilizes the local structure. The zinc binding motif is always located in the vicinity of the catalytic site [21–23] as shown in Fig. 2. Cysteine and histidine residues mostly coordinate the zinc ion with different conformations. Three cysteines and one histidine *versus* cysteine/histidine pairs constitute the *Pyrococcus furiosus* zinc knuckle and the *S. islandicus* zinc stem respectively. An aspartate and three cysteines coordinate the metal in the *Sulfolobus solfataricus* zinc stem [22]. The H141A mutation, which disrupts the zinc, abolishes the pRN1 primase activity [28]. As reported in the study of Lao-Sirieix et al. [17], modifications of the zinc binding motif alters primer synthesis (shorter products). The authors assumed an interaction with the DNA template or NTPs but the exact function of this motif still needs to be determined. It is also noteworthy that bacterial primases have a zinc structure, i.e. the N-terminally located zinc finger whose contribution to primer synthesis is also unresolved. In addition to the structural zinc motif, experimental data showed another important element of functional eukaryotic primases: the iron–sulfur cluster (Fig. 2).

### 2.3 The iron–sulfur cluster

An iron–sulfur cluster is present in the large subunit of eukaryotic primase (Figs. 1 and 2). Biochemical studies have shown the importance of the large p58 subunit supporting p49 in primer synthesis [29–31]. Mutations of conserved cysteines ligating the iron–sulfur center [25,32–34] of p58 completely abolish primase activity. Structures of the C-terminal regions of the human p58 and the yeast PriL proteins reveal novel folds encompassing a solvent-protected 4Fe–4S cluster. The yeast PriL-CTD is similar to the human p58-CTD structure solved in 2010 [34]. Twelve stacked α-helices (PDB ID 3Q36) define two domains (each providing two cysteines) which sandwich the 4Fe–4S center. Surprisingly, the first reported human p58-CTD structure [33] exhibits a two-stranded β-sheet instead of α-helices 4, 5 and 6 in the other structure [35]. This local structural change in the p58-CTD has been proposed to influence DNA binding in the functional assembly. However, the relatively weak affinities measured for the yeast PriL-CTD upon single- and double-stranded DNA binding (100 μM range) contrast with significantly lower K_ds_ measured between the human p58-CTD and the same oligonucleotides [33,35]. Moreover, the human p58-CTD binds the replication protein through the 32 kDa subunit (RPA32) and preferentially to primed DNA [33]. As discussed below PriL-CTD is structurally comparable to DNA photolyase. These enzymes bind single-stranded DNA suggesting that PriL-CTD could also be involved in DNA binding.

### 2.4 The α-helical subdomain

Remarkably all structurally defined archaeo-eukaryotic primases have a α-helical subdomain. The cellular archaeo-eukaryotic small primase subunits have a helical subdomain inserted within the AE_Prim_S domain (Fig. 1). However there is no structural similarity beyond its high helical content. In contrast, the plasmidal primase from pRN1 and RSF1010 have helical subdomains which are highly similar and most interestingly related to the C-terminal part of the PriL-CTD and photolyases [26,28,34]. This might suggest that the latter helical subdomains could carry out an important and conserved task. In fact in the plasmidal primases, the helical subdomain is required for primer synthesis the same as the large primase subunits are required for primer synthesis of the cellular systems.

## 3 Template binding

To date only one structure of a template-bound primase has been reported. Together with NTP binding, interaction with the template is one of the early events of the enzymatic cycle (Fig. 4). The lack of structural information concerning primase/template binary complexes is probably linked to three intrinsic limitations: the single-stranded DNA flexibility, the complexity of the minimal protein assembly required for binding [15] and the relatively weak affinity [28].

### 3.1 The template bound RepB′ primase

**Fig. 5:**
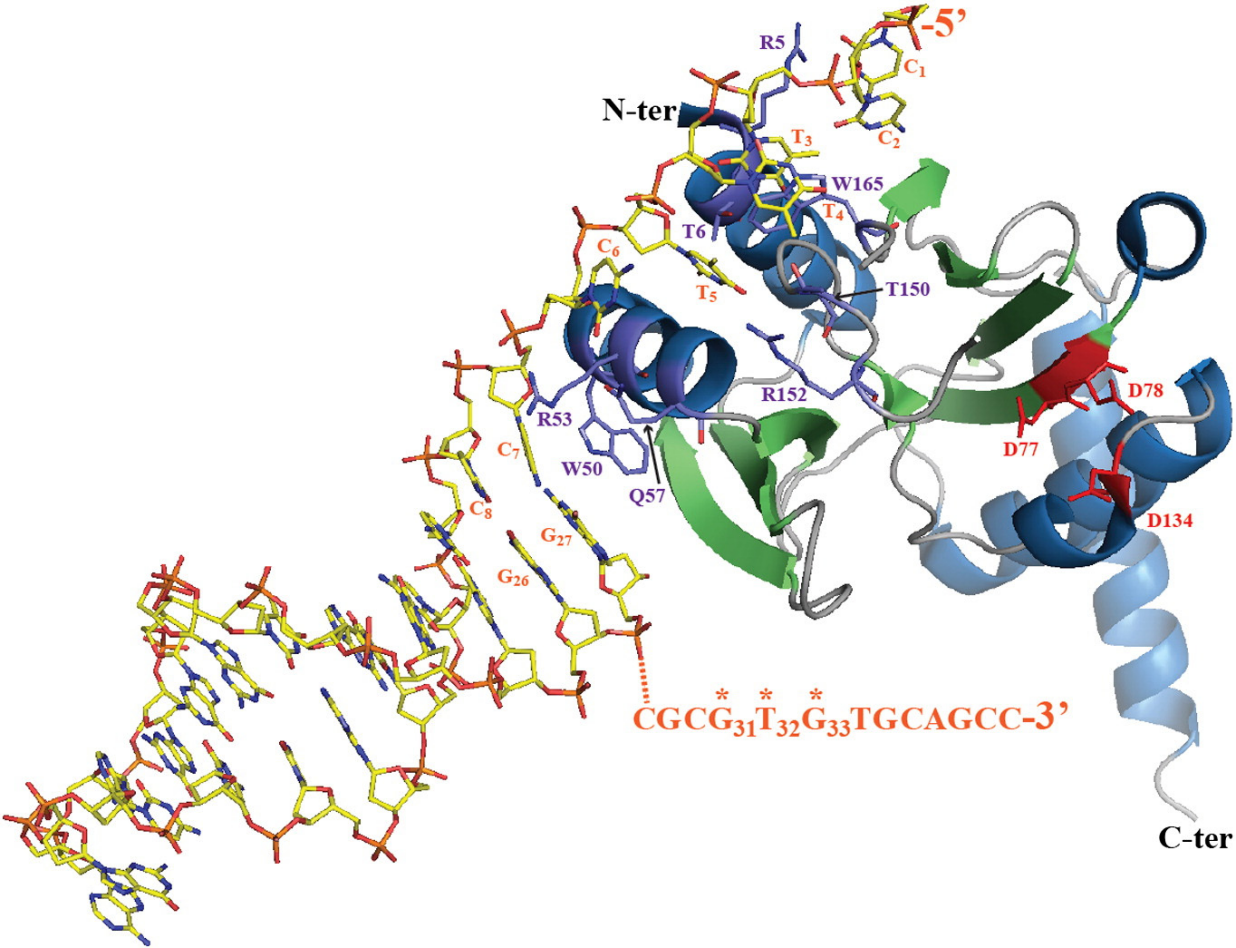
Detailed interaction at atomic resolution between the catalytic domain of RepB′ and the truncated single-stranded initiation site A (*ssiA*). The most important residues (of the RepB′ catalytic domain) contacting the DNA are shown with purple, red and blue sticks for C, O and N atoms respectively. Corresponding residue names are in purple. The solved DNA structure is presented with yellow, red, blue and orange sticks for C, O, N and P atoms respectively. Direct and water-mediated hydrogen bonds as well as stacking interactions are reported between the protein side-chains in contact with the bases and/or the sugar-phosphate backbone of *ssiA* (3′∆13). However, the conserved GTG motif (31 to 33) is located in the 3′-end of the DNA template and the orange dashed line followed by the 13-bases sequence correspond to the missing part. The template-bound enzyme is not in an active conformation which would imply the interaction of the protein with the GTG. The conserved catalytic aspartates (D77, D78 and D134) are displayed with red sticks.

The RepB′ primase of the bacterial plasmid RSF1010 recognizes specific 40 nt-long DNA sequences called initiators: *ssiA* and *ssiB* refer to single-strand initiation sites A and B respectively. Only one *ssi* sequence is present on each plasmid strand and previous works indicated that RSF1010 could be exclusively replicated in the leading-strand mode. The *ssiA* forms a hairpin (nt_7–27_) flanked by 6 (nt_1–6_) and 13 (nt_28–40_) single-stranded DNA sequences in the 5′ and 3′ ends respectively. The RepB′ primase comprises a large catalytic domain (residues 1–205) and a small helix bundle domain (residues 220–323) connected by a 42-aa-long linker (composed of a 28-aa-long α-helix and a 14-aa-long flexible tether). Both domains interact independently with the *ssiA* DNA (2 and 27 μM affinity constants for the catalytic and the helix bundle domain respectively). In the X-ray structure of the template/RepB′ primase complex, the catalytic domain (N-terminus) is bound to the truncated *ssiA* element (*ssiA* 3′∆13) (PDB ID 3H25, [26], Fig. 5). Residues of the catalytic domain establish specific contacts with the single-stranded 5′ tail and the first GC base-pair of the hairpin. Eleven hydrogen bonds connect 9 residues with 7 bases which supports a specific mode of recognition (only 3 contacts with the sugar-phosphate backbone). However, the single-strand segment initiator A lacks 13 nucleotides (3′∆13) including the GTG motif (nt_31–33_) proposed as the initiation site for primer synthesis [26]. Thus this template bound structure does not show the primase in a conformation of dinucleotide synthesis or elongation, but rather a “preparatory” conformation of binding and possibly positioning the template DNA for primer synthesis.

### 3.2 The PriL structure-based DNA template binding model

**Fig. 6:**
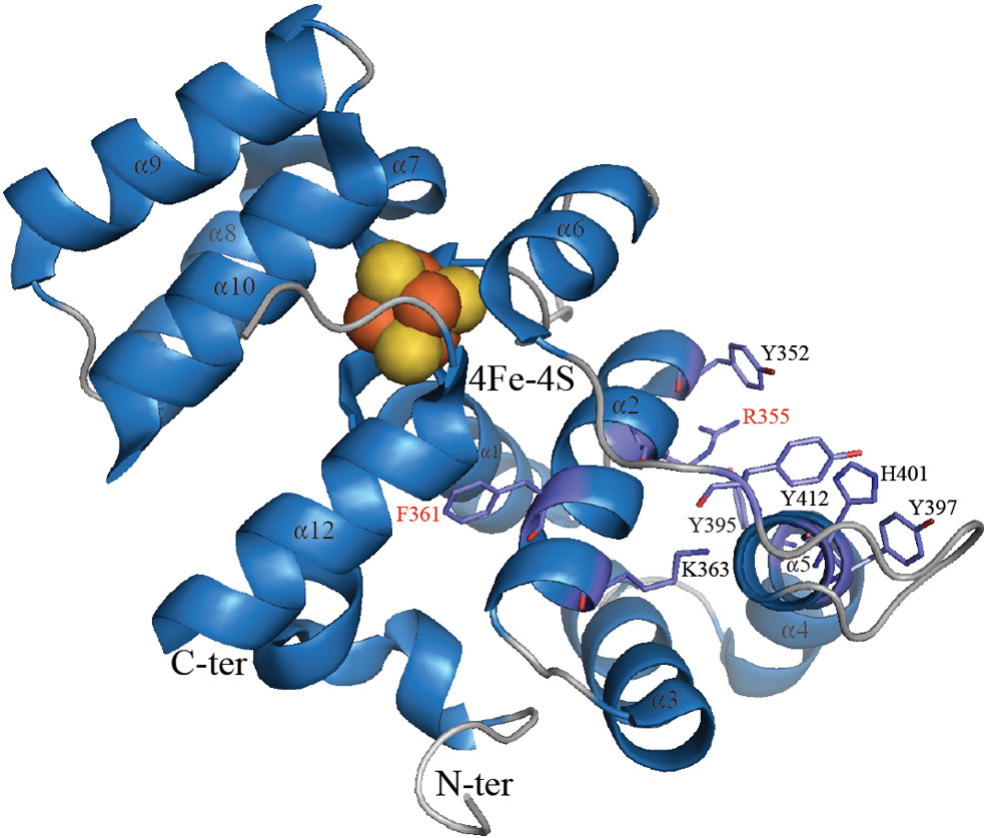
Putative DNA binding interface located on the C-ter part of the large primase subunit from *S. cerevisiae*. The structural similarity between the PriL-CTD (3LGB) and the DNA-bound active site of DASH cryptochrome 3 from *A. thaliana* (2VTB) led Sauguet and collaborators [34] to propose a binding interface for the template. In this model, the large primase subunit (containing the FeS cluster) would assist the small one in the interaction with the DNA template. Key residues potentially involved in template binding are shown with purple, red and blue sticks corresponding to C, O and N atoms respectively. Most of them are located on helices 2 and 5. Invariant amino acids (see structure-based alignment in [34]) are highlighted in red. Orange and yellow spheres of the metal cluster (4Fe–4S) correspond to iron and sulfur atoms respectively. Residues 483 to 494 could not be detected.

The structure of the *Saccharomyces cerevisiae* PriL C-ter domain (PriL-CTD) brought further insights on DNA template recognition by eukaryotic primases. The active site region of the DNA photolyase/cryptochrome family of flavoproteins adopts a similar fold as the N-terminal part of the PriL-CTD [34]. Based on structural homologies between these two elements, authors assumed that this portion of the primase could interact in a similar manner with single-stranded DNA (Fig. 6) as the DNA repair enzymes of the photolyase/cryptochrome family do. Fluorescence anisotropy measurements demonstrated that PriL-CTD binds to 20 nt-long ssDNAs but with an extremely weak affinity (K_D_ of 70 μM). Complementary results corroborate that template binding is mainly carried out by the catalytic PriS subunit. Nevertheless PriL-CTD is proposed to facilitate the positioning of the template relative to the active site of PriS. Moreover, the PriL-CTD protein might favor the dinucleotide/DNA template annealing. Atomic contacts between PriL-CTD and the template DNA can be potentially mediated by patches of highly-conserved basic (2 histidines, 2 lysines and 1 arginine) and polar residues. For example, the conserved H401 of helix 4 could interact with the template phosphate backbone (Fig. 6). Besides direct contacts, a putative DNA-binding loop located between helices 5 and 6 is stabilized by the conserved K363. Obviously, conserved aromatic residues could stack base rings (like tyrosines 352, 395, 397 and 412). As already mentioned above the PriL-CTD helical domain has resemblance with the helical subdomains of the plasmidal primases. Our experiments (data not published) show that the helical subdomain has a strong DNA-binding activity comparable to the phytochrome proteins. In additions, mutations in this part of the protein reduce or abolish primase activity [28] stressing that this helical domain is important for the plasmidal primases and making it plausible that this domain might also be critical for primer synthesis by the cellular eukaryotic primases.

## 4 Nucleotide binding

**Fig. 7:**
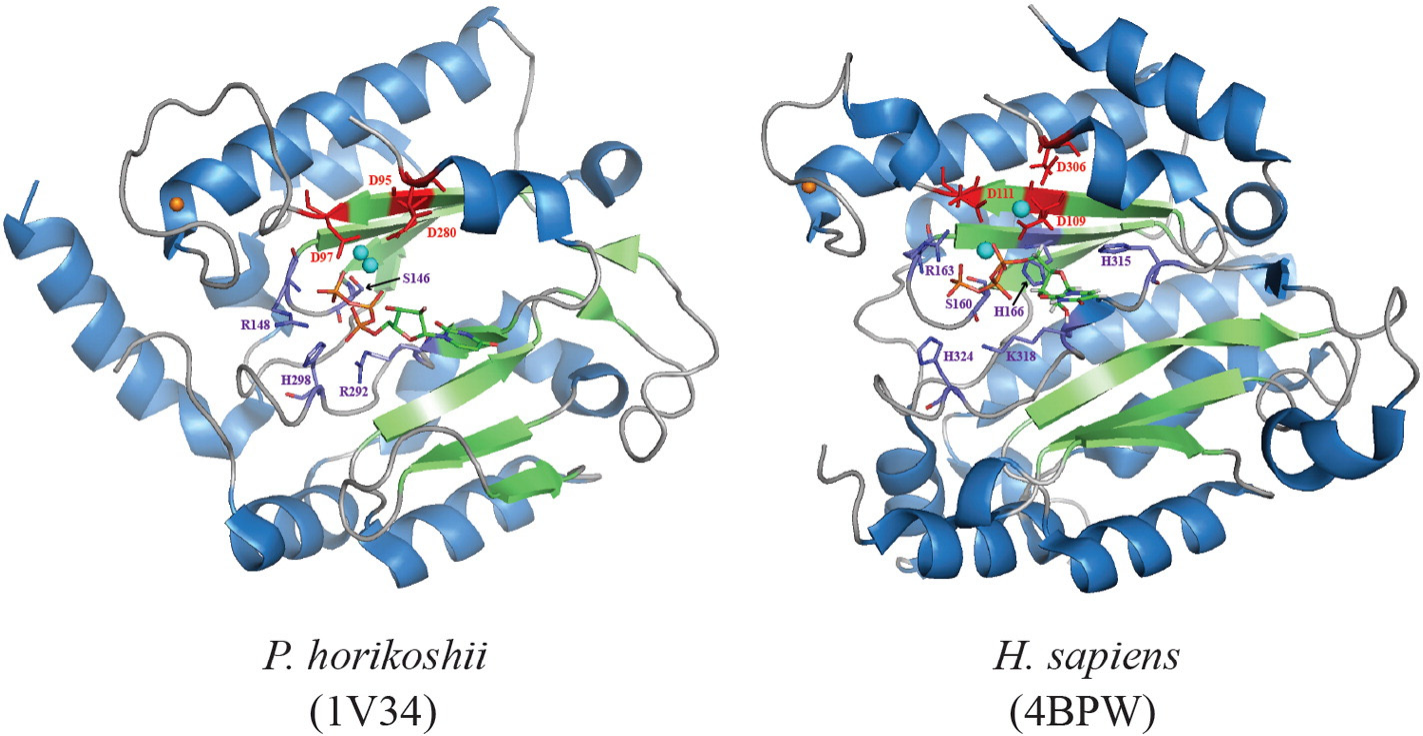
Snapshots at atomic resolution of the UTP-bound catalytic site of two archaeo-eukaryotic primases. Residues backbone and side-chains involved in the interaction between the UTP and the catalytic domain of the archaea (left) and human (right) primases are displayed with purple, red and blue sticks corresponding to C, O and N atoms respectively. Both UTP molecules are shown with green, red, blue, orange and white sticks for C, O, N, P and H atoms respectively. Cyan spheres represent water molecules and manganese atoms in *P. horikoshii* and *H. sapiens* respectively. Conserved aspartates are indicated in red and represented with sticks. Zinc atom is shown with orange spheres. Detailed contacts are described in the “Nucleotide binding” section.

Besides the formation of the template–primase binary complex, nucleotide triphosphates must bind to the enzyme and react to create the first phosphodiester bond. For the eukaryotic/archaea organisms, the snapshot at atomic level of a ternary template–primase–NTP complex is not yet available. However, useful information has been provided by several primase–NTP X-ray structures (Table 1 and Fig. 7). The primase–NTP interaction is limited to the catalytic site of the enzyme and heavily relies on divalent cations' presence. According to biochemical data collected over the years [8], two different NTP-binding sites have been defined: one initiation site for the first nucleotide localized at the primer 5′-end and one pocket required for the incoming NTP. Kuchta and Sheaff [36] demonstrated that the eukaryotic primase first interacts with the incoming nucleotide and then with the initial NTP. In a preliminary assessment, the molecular features of the NTP-protein association are analyzed independently from the pocket type. Then, they will be replaced in the catalysis context.

### 4.1 Nucleotide binding by DNA primases of hyperthermophilic archaea

In 2003, a publication described (at atomic scale) the interaction between an archaeal primase (*Pyrococcus horikoshii*) and UTP in its active site (Fig. 7). By analogy with the two-metal-ion mechanism, enzymatic assays and mutagenesis rapidly connected the highly conserved carboxylate groups in the active site with catalysis. Thus, one metal-ion could contribute to nucleotide attachment and the other one could participate in catalyzing the nucleotidyl transfer [21]. The structure of the UTP-bound *P. horikoshii* primase demonstrated the importance of the three conserved aspartates D95, D97 and D280 which indirectly contact the triphosphate moiety. Although it remained impossible to differentiate magnesium from water molecules at 2.7 Å, a water-mediated hydrogen-bond network partly explains the crucial role of the invariant triad. Indeed, the D95/97 pair, Y150, S146 and the γ-phosphate of the nucleotide can coordinate H_2_O or Mg^2 +^. In the *P. furiosus* enzyme, identical acidic residues can support NTP binding [20]. Notably, in the *P. horikoshii* enzyme without cofactor, two conserved positively charged residues ([R148 and H298] R148, H298) interact with a phosphate ion. In *P. furiosus*, R148 and K300 bind sulfate while in the *S. islandicus* pRN1 prim/pol structure, a sulfate ion is connected to the histidine 141 [28]. In the *P. horikoshii* enzyme, direct contacts have been detected between the triphosphate moiety of UTP and basic amino acids highly conserved among archeo-eukaryotic primases: the α- and γ-phosphate of UTP make hydrogen bonds with the η–NH_2_ and Nε atoms of R148 respectively. Atoms η–NH_2_ and Nε of another arginine, R292, contact respectively the α- and β-oxygen of the triphosphate moiety. Furthermore, the H298 interacts with the γ-oxygen. Unfortunately, the ribose and the base rings were structurally disordered and its detection failed in the electron density map. Authors suggested that the base and the ribose might be stabilized upon DNA template addition. Only one bound UTP molecule is observed in the complex and is probably located in the extension site. The superposition of the active site residues of the *P. horikoshii* primase and the primer/template/nucleotide bound-polymerase β [21,37] performed by Ito and colleagues nicely illustrates the structural ability for the priming enzyme to accommodate a second nucleotide in a potential initiation pocket. The accumulation of three-dimensional data on various NTP–primase binary complexes or solving the quaternary enzyme–template–NTP_initial_–NTP_extension_ structure could definitely help to answer remaining questions. Recent data from eukaryotic primases provided relevant complementary information concerning nucleotide binding.

### 4.2 NTP-bound human DNA primases

**Fig. 8:**
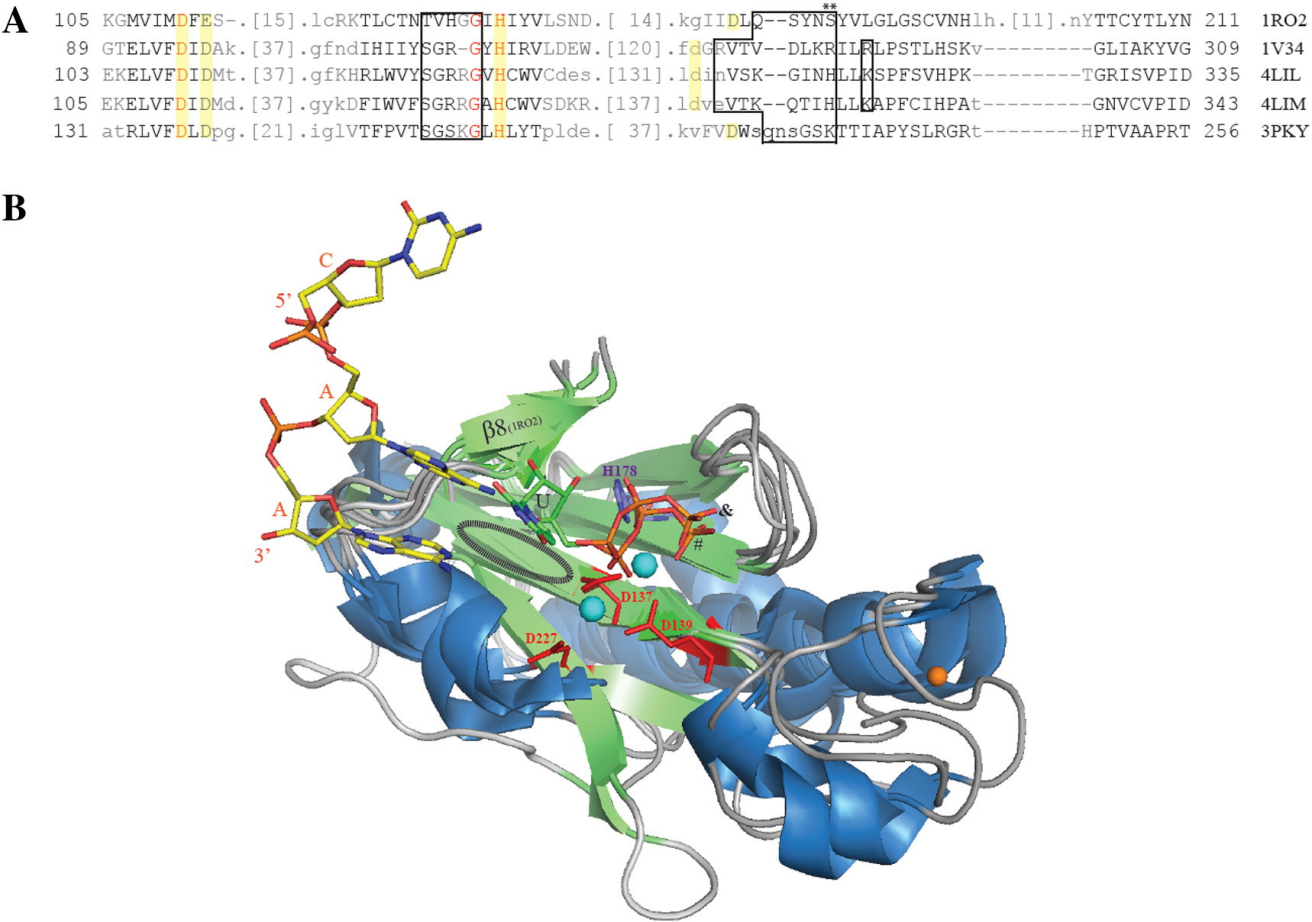
Structure-based sequence alignment and structural superimposition of catalytic cores from the pRN1 (1RO2), *P. horikoshii* (1V34), *H. sapiens* (4LIL) and *S. cerevisiae* (4LIM) primases as well as the polymerase domain of the *M. tuberculosis* ligase D (3PKY). A: The enzymes share three conserved acidic residues and a histidine (yellow background) as catalytic residues. Residues, immediately N-terminal to the catalytic histidine, are important in binding the elongating nucleotide (black box). The position of the initiating nucleotide is less clear as direct evidence is not available. However a “pre-ternary” structure of the polymerase domain of ligase D [40] with the bound elongating NTP and the template DNA suggest that the initiating nucleotide faces the third catalytic aspartate. This presumes that the conserved region C-terminal to this residue (** and small black boxes) is important for binding the initiating nucleotide. Gray, black and red letters correspond to not structurally aligned, structurally aligned and identical residues respectively. Side numbers are the amino acids as referred in the PDB files while the omitted residues are in brackets [ ]. B: The polymerase domain of ligase D has been solved with template and incoming nucleotide. Such a structure is not yet available for primases. We therefore used the polymerase domain of ligase D to investigate the possible positioning of the initiating and elongating nucleotide. Presented are only the catalytic cores of the five proteins. These cores comprise a four-stranded β-sheet (RRM, RNA recognition motif) which harbors two acidic residues and the catalytic histidine as well as the flange running perpendicular above the sheet (labeled β8 in the pRN1 Prim/Pol structure). In atomic resolution are shown the catalytic residues of the polymerase domain of ligase D, i.e. D137, D139, [D277 and H178] D277, H178 as well as the three bases of the DNA template and UTP. The adenine base in the center pairs with the UTP nucleotide, presumably in the elongating position. The initiating base would be positioned next to UTP (black broken ellipse) with its 3′OH group in the vicinity of D227. Its base ring would contact the complementary base of the template. Cyan spheres are the manganese ions which support the catalysis. N-terminal to the flange the secondary structure elements are less conserved but this region harbors a conserved catalytic aspartate residue (D227). & indicates the ligase UTP triphosphate while # corresponds to the triphosphate group of the UTP bound to the catalytic subunit of the human primase (4LIL).

Two structures of human DNA primases upon UTP binding have been released in the last two years [12,25]. Again, only one nucleotide was detected in the presence of magnesium and manganese in the catalytic subunit. Vaithiyalingman and collaborators showed that manganese increased almost 20-fold p48–UTP binding affinity (Kd*_apparent_* = 11 μM). Unfortunately, the structure of the p48–UTP–Mg^2 +^ complex could not be determined (Kd ~ 200 μM). On the contrary, two atoms of magnesium were observed in the active site of the UTP-bound PriS [25] and fortunately, base and ribose rings were visible. Thus, similarly to the archaea primases, conserved acidic and basic residues tether the triphosphate moiety (Fig. 7). The Mg^2 +^-mediated contacts with invariant aspartates (109 and 111) in the β4-strand assist direct interactions of R162 and H166 with γ- and β-phosphate respectively. The third member of the catalytic aspartates (D306) may partly coordinate one magnesium ion. Besides arginines, the serine 160 in the PriS “SGRRG” loop (between β5 and β6) contributes to the triphosphate positioning (see also Fig. 8A). For the binary p48/UTP complex, in the presence of manganese, the side-chain of D109 plays a key role. Indeed, it contacts two manganese ions with the simultaneous engagement of the three phosphates (α, β, γ), the α-oxygen and two water molecules. Furthermore, the conserved p48 basic residues (R162-3, H166, K318) and S160 are directly or indirectly involved in the triphosphate moiety attachment. Authors observed that three of these basic residues (R162/K318/H324) can bind citrate in the unliganded human p48. In the catalytic PriS unit, amino acids of the invariant HLLK motif (315–318) greatly stabilize the ribose and the base rings whereas these fragments remain flexible in p48/UTP structure. Actually, for PriS two backbone atoms (N of L316 and O of K318) are hydrogen bonded to the 2′-OH of the ribose. In addition, one histidine side-chain (H315) may specifically contact the oxygen of the ribose ring. The interaction network is completed by hydrophobic (L316 and 317), electrostatic (K318 side-chain) and water-mediated (H324) interactions. As previously mentioned, the pocket occupied by UTP is plausibly the one for the incoming (i.e. elongating) nucleotide. Kilkenny and coworkers monitored the effect of alanine substitution on the conserved residues surrounding the nucleotide binding site [12]. Interestingly, they argued that two residues (R56 and E44) might be responsible for the 5′-nucleotide binding. For p48 [25], mutagenesis approaches also highlighted the importance of an arginine (R304) which might bind the initiation nucleotide. In order to integrate these structural aspects of primases/nucleotides association in the enzymatic cycle, we discuss and analyze hereunder experimental and *in silico* data related to dinucleotide formation.

### 4.3 Biochemical investigations on nucleotide binding by herpes primase

An informative study published in 2008 focused on the minimal chemical requirement for nucleotides sugar ring binding to the herpes virus primase. This work does not concern an archaea-eukaryotic enzyme but herpes virus and eukaryotic primases discriminate between NTPs in a very similar way [38]. Authors demonstrated that NTP recognition strongly depends on the 5′-γ-phosphate. The use of NDPs or NMPs instead of NTPs strongly compromises binding. Furthermore, they showed that the interaction between nucleotides and the herpes virus primase tolerates significant modifications on the sugar rings. A very important condition for nucleotide binding by herpes primase is the presence of carbon in the 2′ position. Cyclic sugars are preferred but not essential. The omission of the hydroxyl in 2′ and 3′ did not appear to prevent binding even if 2′- and 3′-deoxyribonucleotides are responsible for chain termination. It was also established that the Watson–Crick hydrogen bonding between the NTPs and the template is not critical. This last point can corroborate the low specificity of the herpes and the eukaryotic primases which recognize the template G-Pyrimidine–Pyrimidine and pyrimidines-rich motifs, respectively. More particularly, the effect of NTP analogs on the dinucleotide formation has been monitored. Thus, the synthesis on the first phosphodiester bond is altered when the initiating nucleotide (incorporated in 5′) is a 2′-dNTP or an arabinofuranosyl-nucleotide triphosphate (ara-NTP). This result is independent from the chosen base-ring indicating that the primase does not detect a base-pairing between the template and the 5′-nucleotide. Interestingly, the integration of these two analogs is tolerated during elongation.

## 5 Dinucleotide formation and repositioning at the active site: structure-based models

In the catalytic process required for primer synthesis, it is generally thought that the limiting step is the dinucleotide formation whereas primer extension is relatively fast. The first phosphodiester bond is generated after the assembly of a quaternary complex composed of the primase, the DNA template and the two required NTPs [8]. The nucleotide at the initiation site provides the 3′-OH which can react with the 5′-phosphate of the incoming NTP located in the active site (Fig. 3). The functional analogy with polymerases implies that the conserved residues of primases promote this reaction. Extensive biochemical investigations coupled to structure-based modeling paved the way for the understanding of dinucleotide formation while awaiting the detailed mechanism at atomic scale.

The ternary complex formed by the active site region of the *Arabidopsis thaliana* DASH cryptochrome 3, FAD and a single-stranded DNA displays significant three-dimensional similarities with the C-ter domain of PriL [39]. Sauguet and coworkers suggested that the interactions occurring between the C-ter part of PriL (Fig. 6) with the template and the dinucleotide could resemble the respective recognition modes of single-stranded DNA and FAD mediated by the cryptochrome [34]. However, in order to correlate modeling and experimental data (PriL-CTD binds the DNA weakly) authors claimed that replication initiation requires a synchronized mechanism involving both PriL-CTD and PriS. PriL-CTD may assist PriS with dinucleotide binding. The associated PriS and PriL-CTD would delimitate a pocket to position the dinucleotide and promote its base-pairing with the template.

The UTP-bound p48 binary complex in the presence of manganese [25] contributed to define a model of the eukaryotic primase in its active state comprising the dinucleotide and the template. Indeed, the functional homology highlighted between the X-family DNA polymerase λ and the catalytic site of the p48 primase allows the elaboration of a structure-based snapshot just before phosphodiester bond formation. The DNA polymerase λ structure forms a quaternary complex constituted of a DNA template, a UTP analog and two metal ions (Mg^2 +^ and Mn^2 +^). Besides sequence conservation in the active sites of the two enzymes, the three-dimensional arrangements of the metal ions, the conserved catalytic aspartates and the bound UTPs are very similar. In this model, the initiating nucleotide can be positioned relative to the incoming NTP and to the single-stranded DNA. Interestingly, the involvement of the R304 residue in nucleotide binding [25] at the initiation site suggests a local conformational change. In the X-ray structure of the bound p48 primase, residues in the zinc motif retain R304 and the conserved D111. This topology prevents the recruitment of the NTP in the 5′-end. Thus, significant structural modifications are hypothesized to accommodate the second nucleotide in the closed vicinity of the UTP with a concomitant base-pairing to the recognized sequence. Another relevant model has been provided thanks to the *Pho* primase–UTP complex [21]. In the corresponding work, conserved aspartate residues of the polymerase β (D190, 192 and 256) and the *P. horikoshii* primase (D95, 97 and 280) became reference points for a structural alignment between the catalytic sites. The respective positions of the ddCTP (pol β) and the UTP (primase) triphosphates match quite nicely between the two structures. The polymerase β/template–ddCTP complex shows that the R254 located in the active site can bind the phosphate backbone of the DNA molecule. However, in the primase the bulky arginine side-chain is replaced by a cavity circumscribed by the loop between helices P and Q. Thus, a second NTP might adapt to this pocket. Moreover, hydrophobic neighboring residues (in helix Q) could potentially contact the sugar and the base moiety of the initiating nucleotide.

As shown in Fig. 8, the position of the dinucleotide can also be approximated by using the template and UTP bound to the polymerase domain of the ligase D [40]. This model of dinucleotide formation (based on structural alignment of primases with ligase D) has the advantage that there is structural similarity beyond the catalytic triad as observed for the β/λ DNA polymerases.

After the dinucleotide is formed, the last step of replication initiation is the primer extension. The composition and the length of this short oligonucleotide can vary according to the studied enzyme.

## 6 Dinucleotide extension by archaeo-eukaryotic primases

In the catalytic site, NTPs are successively integrated to extend the dinucleotide to constitute the primer. On average, for eukaryotic DNA primases, the RNA primer length reaches 10 nucleotides. Then, the so-called unit-length primer [36] is transferred to the polymerase α in charge of its elongation [18,36]. The three-dimensional structure of the yeast polymerase α during the elongation process is available [24] but not a complex including the primer, the template and the primase unit in a strict sense. The absence of structures involving the primer, the template and the primase restricts the description of the molecular mechanisms inherent to dinucleotide-primer extension. However, functional analogies with DNA polymerases, enzymatic assays and models can provide tools to compensate the current limitations.

### 6.1 Structurally-related models of dinucleotide extension

The UTP-bound PriS/PriL primase structure released recently and the associated mutagenesis of residues located in the NTP binding site were used to propose a procedure for dinucleotide extension. The conserved residues in the vicinity of the UTP interaction site (but not directly in contact with the cofactor) have been substituted by alanines and the effect on primer synthesis monitored. More particularly, mutation of two charged residues (R56 and E44) totally disrupted primase activity without altering template binding. Hence, they might participate in defining a second NTP-binding pocket. The existence of these two pockets and the fact that all the mutations of conserved residues in the catalytic site affect both dinucleotide synthesis and its extension led authors to presume that the same cluster of residues are involved in both processes. Accordingly, in the proposed mechanism, PriS acts as a template/primer binding platform while the CTD of PriL is repositioned, thereby ensuring the systematic occupancy of the initiation pocket by the 3′-end of the growing primer. It requires a significant degree of flexibility between the two subunits and this has been proposed in a mathematical model in 2011 [41].

### 6.2 A mathematical modeling of primer synthesis

In the work of Ping Xie [41], the eukaryotic p58 unit binds the template whereas the catalytic p49 interacts with the RNA primer. During dinucleotide elongation, the DNA-bound p58 remains fixed while p49 shifts along the template adding NTPs in the 3′-end. The movement of p49 could stretch the linker between the two units which generates an internal elastic force. The affinity of p49 to the primer would increase proportionally to primer length and the improvement of the RNA/protein interaction may somehow drive the catalytic unit progression. When the 9-nt-long primer is synthesized, the maximum distance between residues connecting the two domains is reached and it necessitates molecular rearrangements to translocate the enzyme reinitiating the whole process. Interestingly, when p58/template affinity is impaired, primer production remains weak and its length not controlled [31]. It was also confirmed that the probability of the p58-template dissociation increases after p49 releases the dinucleotide. In addition, the developed analysis considers that p49 could preferentially interact with the RNA in the primer/template duplex. Calculated reaction parameters are even verified if one envisages contacts between p49 and the DNA sugar-phosphate backbone. As reported elsewhere [36], the RNA/DNA duplex may function as both a substrate and an effector. Thus, an allosteric effect provoked by the template–primer interaction with a secondary binding pocket (not identified yet in the primase) might result in the subunits repositioning allowing the transfer and the regulation of DNA synthesis. Mutagenesis analysis and enzymatic tests reinforce the proposed conformational change hypothesis. Selected ones are discussed in the next paragraph.

### 6.3 Complementary data from enzymatic assays

**Fig. 9:**
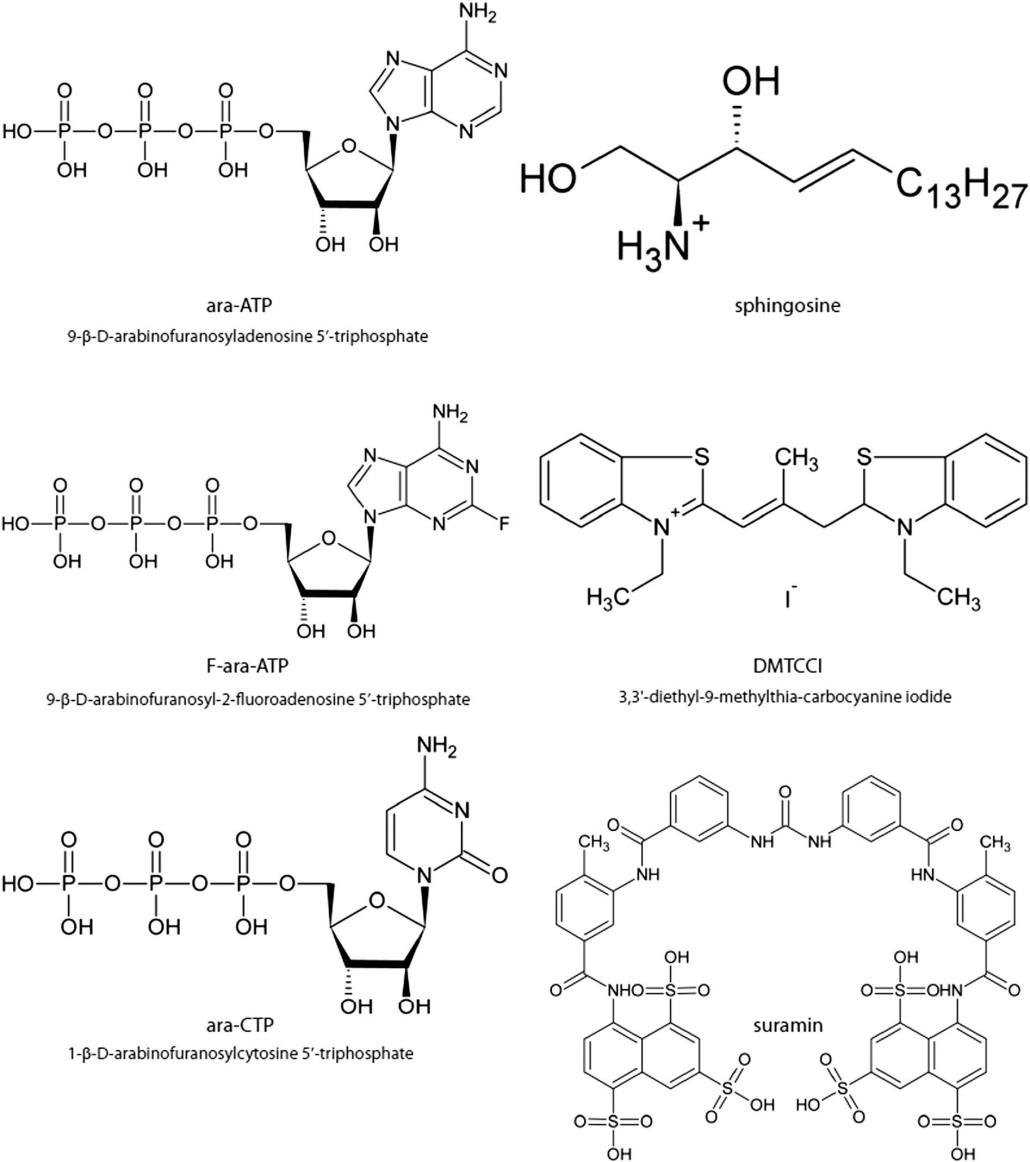
Structures, names and abbreviations of the primase inhibitors mentioned in the text. The systematic names (IUPAC nomenclature) of the NTP analogs and the DMTCCI are specified below their respective abbreviations.

In the pRN1 functional primase system [28], investigations focused on the enzyme lacking the linker (residues [K250 to F260] K250, F260 had no electron density in the crystals) unveiled a small part of the molecular mechanism responsible for dinucleotide elongation. The protein with the deleted linker retains the ability to synthesize the dinucleotide but cannot extend it. In addition to its role in DNA binding the independently structured helix bundle domain contributes to dinucleotide formation. The linker-dependent concerted movement between the two parts might convey the transient dinucleotide/template pair to the catalytic site guiding dNTP addition at the 3′-end. Some interesting information about the requirement for dinucleotide extension is provided by the experiments performed on the herpes primase [38]. For this system, the RNA primer is shortened when modified NTPs are introduced into the reaction mixture. Thus, if 2′-dNTPs replace NTPs, the product length is shorter. Furthermore, modified NTP-riboses (2′,2′-difluoro-2′-, ara and S-ara-NTPs, Fig. 9) reduce primer length to only 4 nucleotides. Modified nucleotides could also act as inhibitors of primase and might be relevant for medical applications.

## 7 Primase as a target for drug development

It was shown that the DNA primase activity difference between the replicating and the static phases in cancer cells is significantly higher compared to this difference in the normal ones [42,43] and targeting the enzyme may have promising applications in anti-cancer drug development [44]. In humans, the small 49 kDa primase subunit is encoded by the *PRIM1* gene located on the 12q13 chromosome. The amplification and the overexpression of the genes in the chromosomal 12q13-15 region are reported in various types of tumors [45]. On the contrary to bacterial and viral (essentially *Herpes simplex*) primases [46–49], a limited number of inhibitors have been studied for the eukaryotic enzymes. The initial compounds identified to inhibit primases are nucleotides analogs, such as ara-ATP or ara-CTP (Fig. 9). These molecules can block the bovine (Ki = 2 μM with ara-ATP) and the human eukaryotic primases (Ki = 122 μM for ara-CTP and 128 μM for ara-ATP) [50,51]. The F-ara-ATP (Fig. 9) exhibits higher inhibitory properties than non-halogenated NTP analogs (with IC_50_ = 2 μM, Ki = 6 μM corresponding to a 25-fold higher affinity for F-ara-ATP than ATP) [52]. DNA primases incorporate 30-fold more efficiently the F-ara-ATP into RNA primers than ATP. Previous analysis showed that F-ara-ATP is a non-competitive inhibitor of primer synthesis. Once inserted to the RNA primer, it plays the role of a chain terminator [53]. However, nucleotide analogs suffer from a major drawback since these molecules also impede other enzymatic processes in nucleic acid metabolism (DNA polymerases, RNA polymerases) at concentrations lower than those required for DNA primase inhibition [54]. Non-derived NTP compounds, like suramin [55] (Fig. 9), are suspected to hinder the priming reaction. The Yoshida group has shown that sphingosine (Fig. 9) could compete with DNA upon primase binding and could significantly alter enzymatic activity (90% of inhibition at 4 μM) [54,56,57]. Notably, it does not affect polymerases α or β [56]. Moreover, in the presence of sphingosine the cell growth of human leukemia cells slows down [54]. The three-dimensional analysis of the bound sphingosine (or its analogs) could serve as a starting point for structure-based design of promising DNA primase inhibitors [57]. Finally, the inhibition of DNA primase in hepatocellular carcinoma cells by the DMTCCI molecule (Fig. 9) has been described [44]. The IC_50_ value was 162 nM (inhibitory rates of 80% with 1 μM of DMTCCI). The study demonstrated that DMTCCI has an effect on cell growth of carcinoma cell (IC_50_ = 2 μM) [44]. Although some compounds present an interesting inhibitory potential, no clinical investigations with anti-cancer drugs targeting DNA primases have been reported yet. Indeed, the short length of the synthesized products and the slow rate of primases may be elements that could explain the limited development of high-throughput screening approaches classically adopted for drug discovery. Given the large structural differences between bacterial and eukaryotic primases (see Section 1.1), bacterial primases might also represent valuable targets for antibacterial compounds. It can be expected that compounds targeted to bacterial primases will not inhibit eukaryotic primases. Thus these compound might be used to combat bacterial infections which cannot be treated with conventional antibiotics [58].

## 8 Concluding remarks: What is missing?

Much relevant structural data have been already collected on different archaeo-eukaryotic primases (12 publications between 2001 and 2014, Table 1). Domain topologies of the unliganded enzyme progressively participate in the development of models to describe the priming reaction. Then, binary complexes with NTP molecules (mainly associated with manganese) have improved our knowledge concerning the key step of primase function *i*.*e*. the formation of the first phosphodiester bond. However, additional high-resolution structures are expected to uncover the entire mechanism of replication initiation. We defined a ranking of the desired components. First, a quaternary complex of primase, both nucleotides and the template would be of high interest, as this structure could explain how the dinucleotide synthesis is carried out. This crucial step of the primase reaction cycle is not yet well defined and could confirm or refute whether the reaction proceeds analogously to the two-metal catalyzed elongation step in DNA polymerases. Such a structure would define the exact localizations of the initiation and the elongation pockets. Secondly, a ternary complex including the enzyme, the template and the base-paired dinucleotide would also represent an important structure which could demonstrate how the active site and especially the initiating nucleotide binding sites are reorganized upon repositioning. Thirdly, a very interesting structure would involve the primase in interaction with the primer/template duplex. It may not contribute to fully understand the first phosphodiester bond formation but it might confirm the relative repositioning of the subunits. Finally, full or partial assemblies of the different eukaryotic primase subunits with or without partners (DNA polymerase α, RPA etc.) could help to place the priming machinery in a broader biological context and would probably require electron microscopy based approaches. Obviously, deciphering the primase action with only a mechanistic approach cannot be achieved, structural snapshots at the atomic scale of the active enzyme are absolutely necessary. Thus, pursuing the efforts in further biophysical investigations would also expand fundamental comprehension of the reaction.

## Figures and Tables

**Table 1:** Summary of the most important high-resolution structures mentioned in the analysis.

Protein	Organism	Associated metals	Cofactors	PDB ID	Comments	References
Primase	Plasmid pRN1 *Sulfolobus* *islandicus*	Zn		3M1M	Residues (res.) 36–370	[28]
Primase	Plasmid pRN1 *Sulfolobus* *islandicus*	Zn, Mn		1RO2	Triple mutant	[23]
Primase	Plasmid RSF1010		Single-stranded initiator DNA	3H25	Res. 1–212 27 nt DNA	[26]
Primase	*Pyroccocus* *furiosus*	Zn		1G71	Res. 1–344	[20]
Primase	*Pyroccocus* *horikoshii*	Zn	Uridine 5′-triphosphate	1 V34	Res. 1–346	[21]
Primase	*Sulfolobus* *solfataricus*	Zn		1ZT2	PriS: res. 1–330 PriL: res. 1–212	[22]
Primase	*Saccharomyces* *cerevisiae*	Zn, 4Fe		3LGB	PriL-CTD: res 316–512 Fe–S cluster	[34]
Primase	*Homo sapiens*	Zn, Mn	Uridine 5′-triphosphate	4LIL	p48: res. 1–390	[25]
Primase	*Homo sapiens*	Zn		4LIM	p48: res. 8–396	[25]
Primase	*Homo sapiens*	4Fe		3Q36	PriL res. 266–457 Fe–S cluster	[35]
Primase	*Homo sapiens*	Zn, Mg	Uridine 5′-triphosphate	4BPW	PriS: res. 1–420 PriL: res. 1–253	[12]
Primase	*Homo sapiens*	Zn		4RR2		[60]
Polymerase α	*Saccharomyces* *cerevisiae*		RNA primer DNA template	4FXD	Res. 349–1258 16 nt DNA 10 nt RNA	[24]
Polymerase α	*Saccharomyces* *cerevisiae*		2′-deoxyguanine 5′-triphosphate RNA primer DNA template	4FYD	Res. 349–1258 25 nt DNA 12 nt RNA triple mutant	[24]
Putative DNA ligase-like protein	*Mycobacterium tuberculosis*	Mn	Uridine 5′-triphosphate DNA	3PKY	PolDom res. 6–291 5 + 5 nt DNA	[40]
Cryptochrome DASH	*Arabidopsis thaliana*		FAD* MHF^#^	2VTB	Res. 44–569 5 nt DNA	[61]

We listed the protein type, the organism, the PDB codes and the corresponding references. In addition, we indicated the protein boundaries and the oligonucleotides length (if required) as well as the associated metals and cofactors. *FAD and ^#^MHF are Flavin-Adenine Dinucleotide and 5,10-Methenyl-6,7,8-Trihydrofolic acid respectively. Empty squares in the “Associated metals” column indicate that no metal atoms are present in the PDB file.
